# Balloon-Occluded Transcatheter Arterial Chemoembolization (b-TACE) for Hepatocellular Carcinoma Performed with Polyethylene-Glycol Epirubicin-Loaded Drug-Eluting Embolics: Safety and Preliminary Results

**DOI:** 10.1007/s00270-019-02192-y

**Published:** 2019-03-06

**Authors:** Pierleone Lucatelli, Luca Ginnani Corradini, Gianluca De Rubeis, Bianca Rocco, Fabrizio Basilico, Alessandro Cannavale, Pier Giorgio Nardis, Mario Corona, Luca Saba, Carlo Catalano, Mario Bezzi

**Affiliations:** 1 Vascular and Interventional Radiology Unit, Department of Diagnostic Service, Sapienza University of Rome, Viale Regina Elena 324, 00161 Rome, Italy; 2 Department of Medical Imaging, Azienda Ospedaliero Universitaria (A.O.U.) of Cagliari-Polo di Monserrato, Cagliari, Italy

**Keywords:** Transcatheter arterial chemoembolization (TACE), Balloon-occluded transcatheter arterial chemoembolization (b-TACE), Drug-eluting microsphere transarterial chemoembolization (DEM-TACE), Balloon micro-catheter, Hepatocellular carcinoma (HCC), Safety profile

## Abstract

**Purpose:**

To report technical success, safety profile and oncological results of balloon-occluded transcatheter arterial chemoembolization using a balloon micro-catheter and epirubicin-loaded polyethylene-glycol (PEG) microsphere (100 ± 25 µm and 200 ± 50 µm) in patients with hepatocellular carcinoma (HCC).

**Materials and Methods:**

This is a single-centre, single-arm, retrospective study with 6-month follow-up. Twenty-two patients (Child–Pugh A 68% [15/22], B in 32% [7/22]; age 67.05 ± 14 years) with 29 HCC were treated in 24 procedures. Technical success is defined: ability to place the balloon micro-catheter within the required vascular segment, balloon-occluded arterial stump pressure drops and assessment of microsphere deposition. Laboratory assessment pre/post-procedural and complications were analysed, respectively, according to Common Terminology Criteria for Adverse Events (CTCAEv5) and CIRSE system. Postembolization syndrome (PES) was defined as fever and/or nausea and/or pain onset. Oncological results were evaluated using m-RECIST criteria with CT/MRI imaging at 1 and 3–6 months. In partial responder patients, pre/post-procedural tumour volume was compared.

**Results:**

Pre-planned feeder was reached in all cases. Pressure drop average was 51.1 ± 21.6 mmHg. Exclusive target embolization was achieved in 14/24 procedures (58.3%). Laboratory test modifications were all grade 1. 4/24 adverse events occurred (17%): pseudo-aneurysm of the feeder (grade 3), liver abscess (grade 2) and 2 asymptomatic segmentary biliary tree dilatations (grade 2). PES occurred in 8/24 (33%). The complete response at 1 and 3–6 months was 44.8% (13/29) and 52.9% (9/17), respectively. The partial response at 1 and 3–6 months was 55% (16/29) and 4/17 (23.5%), respectively. Among partial responder patients, the average percentage of tumour volume reduction was 64.9 ± 27.3%.

**Conclusion:**

Epirubicin-loaded PEG microsphere b-TACE is technically feasible, safe and effective procedure for HCC treatment.

## Introduction

Transarterial therapies represent the standard of care for intermediate hepatocellular carcinoma (HCC) not amenable to curative treatments and an option in early stage if surgery/ablation was contraindicated [1]. Balloon-assisted TACE (b-TACE) that is a TACE procedure performed with a balloon micro-catheter inflated within the lesion’s arterial feeders prior to the embolization has been recently developed in Japan by Irie et al. [2]. Balloon micro-catheter intervention works by way of blood flow modification, through a drop of intra-arterial pressure that provides a re-distribution of the flow towards areas of less resistance, in this case tumour lesions [3]. Safety and efficacy of the b-TACE procedure with lipiodol in combination with anticancer drugs have been recently confirmed by some reports [4–6]. Several retrospective studies comparing oncological results on patients treated with b-TACE with lipiodol, with those of an historical matched cohort treated with c-TACE, demonstrated that b-TACE may obtain better tumour control over c-TACE in tumours up to 4 cm [4, 7, 8].

Notwithstanding these advantages, it is to note that lipiodol conventional TACE (c-TACE) has shown several limitations (procedure standardization, toxicity profile, pain), overcome by the introduction of drug-eluting microsphere transarterial chemoembolization (DEM-TACE) [9–11].

On the basis of those results, the combination of DEM-TACE with the b-TACE may improve the oncological response in HCC. The choice of a different delivery system will provide the Interventional Oncologist with another opportunity to further address the specific needs of every single patient, in line with the modern concept of individualized medicine. To date, the combined use of these two techniques has not been reported.

The aim of this study is to describe our experience with b-TACE performed with polyethylene-glycol epirubicin-loaded drug-eluting embolics in HCC patients and report about the safety and preliminary oncological results at 3- and 6-month follow-ups.

## Materials and Methods

This study was approved by the ethical institutional review board. Informed consent for the procedure for anonymized publication of this series of patients was obtained from all individual participants included in the study.

Between January 2018 and May 2018, we treated 22 consecutive patients (mean age 67.1 ± 14.0 years [range, 41–86 years]; 20 males) with 29 hepatocellular carcinoma (HCC) (average 1.3 HCC nodule/patient) [12, 13]. All patients were evaluated by a multidisciplinary board (composed of a transplant surgeon, an interventional radiologist, body radiologist and a hepatologist).

Inclusion criteria were: Child–Pugh score up to B8 and Barcelona Clinic Liver Cancer (BCLC) stage up to B and not eligible for curative treatments (surgical resection or percutaneous ablative treatments). Patients presenting with Child–Pugh > B8, BCLC stage C, portal vein thrombosis (defined as the complete or partial obstruction of blood flow in the portal vein, due to the presence of a chronic, acute or neoplastic thrombus in the vasal lumen) [14], extrahepatic metastasis, and high-flow arterioportal or arteriovenous shunts, previous systemic treatment, platelet count < 50,000, and bilirubin level > 3 mg/dL, were not considered suitable for the procedure.

Diagnosis of HCC was performed according to the American Association for the Study of Liver Disease guidelines [13], using multidetector computed tomography (MDCT) (14/22 patients [64%]) or contrast-enhanced magnetic resonance imaging (CE-MRI) (8/22 patients [36%]).

Clinical and demographics characteristics of the cohort as well as the indications for treatment are shown in Table 1.

**Table 1:** Demographic characteristics

Patient number; nodule number	*N *= 22; *N *= 29
Number of TACE performed	*N *= 24
Sum of diameters: < 3 cm	*N *= 16
3–5 cm	*N *= 6
> 5 cm	*N *= 7
Nodules dimension	
Maximum diameter. mm. (mean value ± SD. range)	32.1 ± 14.4 (12–64)
Minimum diameter. mm. (mean value ± SD. range)	26.0 ± 13.0 (9–53)
Nodules’ volume sum. cm^3^ (mean value ± SD. range)	22.8 ± 27.6 (0.8–101.9)
Age, year (mean value ± SD. range)	65.1 ± 14.8 (41–86)
Sex (M/F)	18/4
Child–Pugh *N* (%)	
A5	13 (59%)
A6	4 (18%)
B7	3 (13.6%)
B8	1 (4.5%)
B9	1 (4.5%)
BCLC *N* (%)	
A	9 (41%)
B	12 (55%)
C	1 (5%)
Aetiology: *N* (%)	
HCV	12 (55%)
HBV	6 (27%)
Alcohol-related cirrhosis	1 (5%)
Cryptogenetic cirrhosis	1 (5%)
NASH	2 (10%)
MELD: *N* (%)	
< 10	11 (50%)
≥ 10	11 (50%)
MELDNa: *N* (%)	
< 10	11 (50%)
≥ 10	11 (50%)
Mono-focal *N* (%)/multi-focal disease *N* (%)	9 (41%)/13 (59%)
Mono-lobar/multi-lobar disease (*N*)	11 (50%)/11 (50%)
AFP serum level	
< 7 μg/L	10 (45.4%)
7–400 μg/L	7 (31.8%)
≥ 400 μg/L	5 (22.8)
Indications for b-TACE	
Down-staging	7 (31.8%)
Bridging	4 (18%)
Palliative	11 (50%)

*TACE* Transarterial chemoembolization; *SD* standard deviation; *M* male; *F* female; *HCV* hepatitis C virus; *HBV* hepatitis B virus; *NASH* non-alcoholic steatohepatitis; *MELD* model for end-stage liver disease; *AFP* α- fetoprotein

### B-TACE Technique and Outcome Measurements

All procedures were performed through right femoral access under local anaesthetics. Liver tumour vascularization map and lesion’s feeder detection (number and site) were obtained with digital subtraction angiography (anterior–posterior and right anterior oblique 25°) and dual-phase cone-beam computed tomography (CBCT) performed through a 4-Fr diagnostic catheter positioned in the common hepatic artery. Based on the information obtained by these two imaging modalities, the best location for the balloon micro-catheter was identified, which is proximal to all lesions’ feeder.

The balloon micro-catheter (Occlusafe, Terumo Europe NV, Leuven, Belgium) is 2.8 Fr micro-catheter with an occlusion balloon on the tip. The micro-balloon is made of compliant polyurethane and is 10 mm in length. (The balloon location is indicated by two radiopaque markers.) The diameter ranges from 1 to 4 mm, according to the volume injected. The micro-catheter can be inserted within a standard 4 Fr angiographic catheter with a co-axial technique. The balloon micro-catheter works on a 0.014’’ platform; in this series, all procedures were performed using a hydrophilic guidewire (GT guidewire, Terumo Europe NV, Leuven, Belgium). Micro-balloon inflation was done with a solution of 1:4 of contrast media/saline.

Once the balloon micro-catheter was positioned within the target vascular segment, the arterial pressure at the tip of the micro-catheter was measured, using an invasive arterial pressure measurement kit. Thereafter, the balloon was inflated to occlude the flow and to obtain a drop of the balloon-occluded arterial stump pressure (BOASP).

The embolization was then performed, according to our routine clinical practice, with drug-eluting microsphere (Lifepearl, Terumo Europe NV, Leuven, Belgium [100 ± 25 µm and 200 ± 50 µm]) [15]), pre-loaded with 50 mg epirubicin per syringe (2 syringes).

Embolization was performed according to the following protocol: the smaller particles (100 ± 25 µm; loaded with 50 mg of epirubicin) were injected first. If the embolization end-points were not reached, the larger particles (200 ± 50 µm; loaded with 50 mg of epirubicin) were then injected.

The embolization *outcome measurement* was composite due to the presence of the inflated balloon micro-catheter that prevented evaluation of flow stasis. Saturation of tumour vascular bed during B-TACE was indicated by the following embolization *outcome measurements*: reflux of microsphere despite balloon inflation following forced injection (2/24 cases [8%]), visualization of vascular anastomosis that could determine potential non-target embolization (1/24 cases [4%]), and manual perception of resistance to embolic injection (10/24 cases [41%]).

The procedure was stopped once at least one of the *outcome measurements* was met or the maximum threshold of epirubicin (11/24 cases [45%]), and DEM had been injected (respectively, 100 mg epirubicin and 2 vails of DEM).

Finally, at the end of the embolization, a non-enhanced CBCT was performed, to evaluate the DEM deposition using a qualitative modification of the categorization proposed by Wang et al. [16]. The evaluation was performed as follows: complete deposition of DEM in the tumour defined as qualitative tumour contrast retention (qTCR) and qualitative defect of tumour contrast retention (qDCR). The qDCR parameter was further distinguished into qDCRa and qDCRb, by correlating the non-enhanced post-procedural CBCT with the pre-embolization arterial phase of the CBCT. When the deposition defect was correlated with the presence of a feeder that was not embolized during the procedure, this was classified as qDCRa, whereas qDCRb indicated a deposition defect not related with the presence of a non-embolized feeder but to a suboptimal DEM intra-tumoural deposition (probably due to no homogeneity of internal tumour architecture). Moreover, the presence/absence of DEM deposition within the surrounding healthy liver parenchyma was assessed and classified using a 4-point Likert scale (0: absence, 1: minimal; 2: mild; and 3: diffuse). The deposition analysis was performed by two Interventional Radiologists (BLIND and BLIND 10 and 5 years of experience) in consensus.

The b-TACE selectivity was categorized according to a dedicated score. (The score is described in detail in Table 2.)

**Table 2:** Embolization score

Score	Description	Procedure (*n* [%])
0	Super-selective	4 [16.6%]
1	Sub-segmental artery	6 [25%]
2	Segmental artery	8 [33.3%]
3	Multi-segmental	5 [20.8%]
4	Lobar	1 [4.1%]

### Imaging Follow-Up Timeline

Follow-up imaging was performed at 1 and 3–6 months from b-TACE procedures with MDCT/CE-MRI and evaluated using the modified Response Evaluation Criteria in Solid Tumours (m-RECIST) [17] by an expert abdominal radiologist, not involved in the procedure.

### Study Outcome Measurements

Primary *outcome measurements* were the technical success and the safety of the procedure.

Technical success was defined as a composite *outcome measurement*: ability to place the balloon micro-catheter inside the required vascular segment, drop of balloon-occluded arterial stump pressure (BOASP) and qualitative assessment of DEM deposition in the target tumour. Moreover, BOASP was correlated with the tumour volume analysis and diameter (< 3 and > 3 cm).

The safety of the b-TACE using DEM loaded with epirubicin was evaluated. In particular, adverse events (AEs) were recorded, and liver function tests and the routine laboratory examinations were analysed before and after the procedure (within 36 h). The AEs were categorized according to the CIRSE classification system [18]. The laboratory test modifications, pre- and post-procedure, were evaluated according to the Common Terminology Criteria for Adverse Events (CTCAE) v5.0 [19]. Post-embolic syndrome (PES) was defined as the onset of post-procedure fever and/or nausea and/or pain (pain score > 6 on a visual analogue scale (VAS) and evaluated prior to discharge. Asymptomatic liver bile duct injuries (ALI) were defined as segmental duct dilation, biloma formation, eventually associated with jaundice [20]. Liver function tests included: serum total and conjugated bilirubin, aspartate aminotransferase (AST), alanine aminotransferase (ALT), alkaline phosphatase (ALP), gamma-glutamyl transferase (GGT) and albumin level. The routine laboratory examinations included full blood count and the coagulation profile (international normalized ratio [INR]).

The secondary *outcome measurement* was the oncological outcome, evaluated at 1 month and 3–6 months, according to m-RECIST criteria [17] at second line imaging (MDCT or CE-MRI). The analysis was performed on a per-patient and also on a per-procedure basis; tumour’s volume modification between baseline (pre-procedure) and post-procedural (1 month and 3–6 months) was employed to stratify those patients who experienced partial response.

The volume was measured by manual segmentation of tumour margins per single slice (1 mm of slice thickness for MDCT and 3 mm for CE-MRI). Measurements of the volumes were obtained twice in order to minimize measurement error.

### Statistical Analysis

The Kolmogorov–Smirnov *Z* test was performed to assess normality distribution for all variables tested. Continuous normal variables were expressed as mean ± standard deviation. Continuous non-normal variables were expressed as median and confidential interval (CI) 95%. The Student T test for paired sample was used for those with normality distribution, and the Wilcoxon rank-sum test for non-normal distribution. Pearson’s test and partial correlation test were performed to assess the relation between BOASP and tumour volume analysis. Statistical analysis was performed, and the graph was plotted using MedCalc 8.0 software (MedCalc, Mariakerke, Belgium). *P* values < 0.05 were considered statistically significant, and all *P* values were calculated using a two-tailed significance level.

## Results

**Fig. 1:**
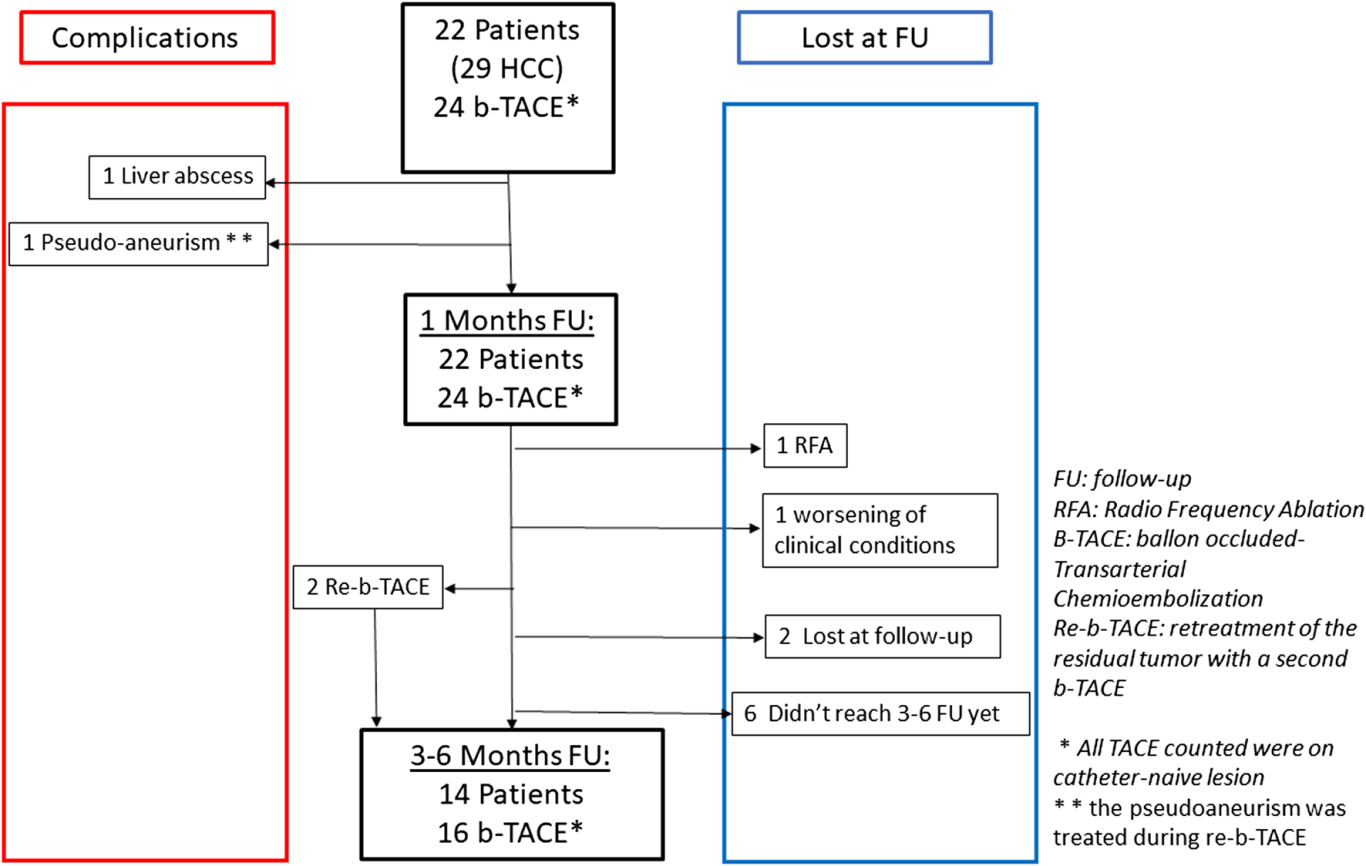
Flow chart showing cohort follow-up details

Twenty-four b-TACE procedures were performed in 22 patients with 29 HCC. Patients’ indication for b-TACE was down-staging to liver transplantation in 7/22, bridging to liver transplantation in 4/22 and tumour palliation in 11/22. Two patients received a second b-TACE for treating a different HCC lesion fed by a different arterial feeder, whereas in 2 cases a second b-TACE (re-b-TACE) was performed for treating the residual vital tumour, either within the same catheter positioning or within another feeder. One patient received radiofrequency ablation (RFTA) of the residual viable tumour after 1-month follow-up. (Details on cohort follow-up are shown in Fig. 1). All these procedures were performed within 30 days of the CT scan performed 30 days after the first procedures.

All 22 patients fulfil the 1-month follow-up. Of 22 patients, 14 (63.6%) performed the 3–6-month follow-up, particularly 6/8 (75%) did not perform the second line imaging modality yet, 1/8 (12%) follow-up not performed for worsening of clinical condition, and 1/8 (12%) underwent to RFTA (Fig. 1).

The technical success of the procedure was 100%. Planned location within the feeding artery was reached in all cases. (The selected arteries expressed as the selectivity score are summarized in Table 2).

The BOASP with the micro-balloon inflated was 64.0 ± 15.0 mmHg (min 46; max 110 mmHg), while prior to inflation it was 115.4 ± 29.5 mmHg; therefore, the pressure drop average was 51.5 ± 21.5 mmHg. An inverse correlation was found between BOASP drop and tumour volume reduction (*r *= − 0.45, *P *< 0.05), and this inverse correlation remained significant considering the pre-procedural diameter of the tumour (*r *= − 0.45, *P *= 0.04).

**Fig. 2:**
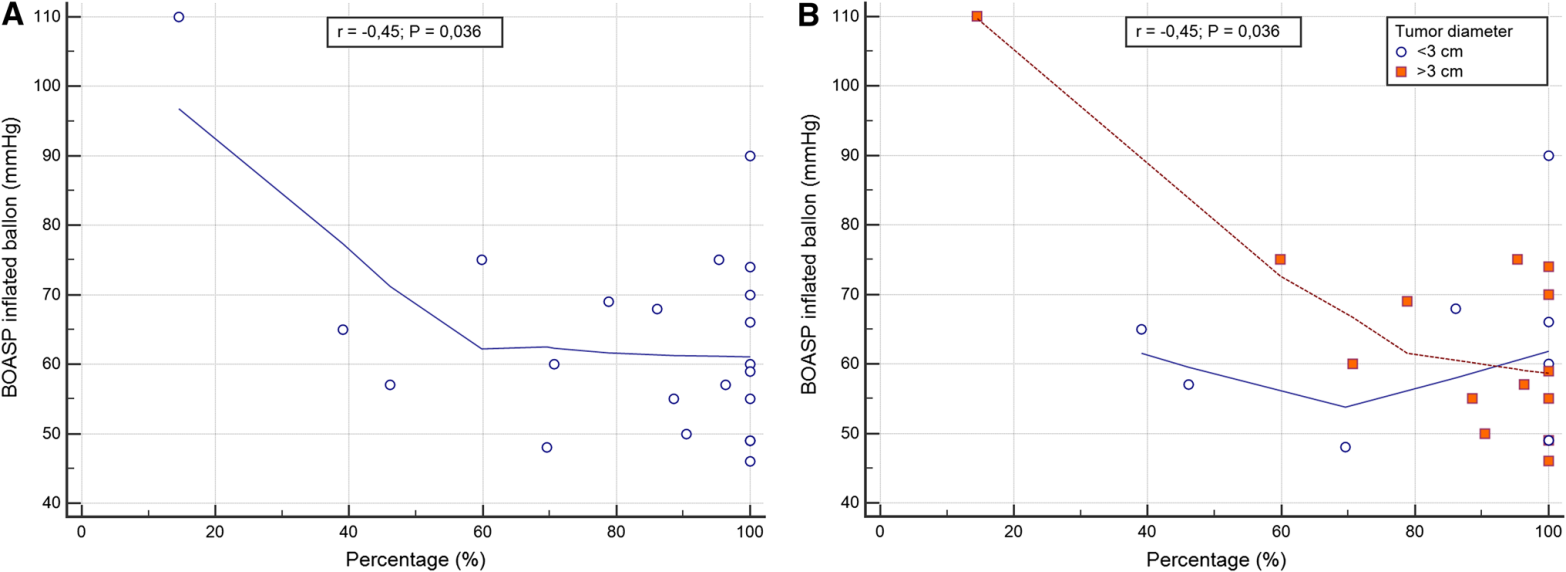
Correlation analysis of balloon-occluded arterial stump pressure (BOASP) and tumour debulking. Figure **A** shows the inverse correlation between the BOASP and the debulking percentage; these data remained statistically significant when tumour was stratified for diameter (< 3 cm and > 3 cm) as highlighted in figure **B**)

The subgroups (diameter > 3 cm or < 3 cm) correlational analysis showed no differences between the two curves (*r *< 3 cm = − 0.05 and *r *> 3 cm = − 0.46; *P *> 0.05) as summarized in Fig. 2. DEM deposition showed qTCR in 14/24 (58.3%); qDCR was observed in 10/24 (41.7%), qDCRa in 2/10 (20.0%), and qDCRb in 8/10 (80.0%). In 13/24 (54.2%) of the procedures, we observed an extra-nodule DEM deposition, among these 1/13 (7.7%) was minimal (grade 1), 8/13 (61.5%) was mild (grade 2), and 4/13 (30.8%) was diffuse (grade 3).

The median epirubicin dose delivered was 62.5 mg [95% CI 55.0–100.0], the median percentage of 100 ± 25 µm microsphere administered was 100% [95% CI 100.0–100.0] (21/24 [88%] completed vial of 100 ± 25 µm), while the median percentage of 200 ± 50 µm infused was 22.5% [95% CI 7.5–100.0] (8/24 [33%] completed vial of 200 ± 50 µm).

Four AEs (15%) were reported: one pseudo-aneurysm of the inflated artery (maximum diameter 5 mm; complication grade 3), one liver abscess (maximum diameter 42 mm; complication grade 2) and two ALI [asymptomatic segmentary biliary tree dilatation (complication grade 2)].

The pseudo-aneurysm was treated using detachable coils (Concerto™, Medtronic, USA), without sequelae. The liver abscess was treated using antibiotic therapy only. The ALI was observed in a patient after a re-b-TACE.

After 8/24 (33.3%) procedure, patients experienced PES. In detail, 5/24 (20.8%) had abdominal pain (VAS score 6.7 ± 1.2), 5/24 (20.8%) had nausea, and 1/24 (4.2%) had low-grade fever.

Statistically significant increase of laboratory test was observed for ALT, AST, direct bilirubin, neutrophil percentage and white blood cells (for details, see Table 3). All changes were grade 1 according CTCAEv5. On the other hand, ALP and total bilirubin significantly decreased when comparing pre- and post-procedure laboratory results.

**Table 3:** Laboratory tests before and after balloon-occluded TACE

Parameters	Lab-pre (average ± SD/median [95% CI])*	Lab-post (average ± SD/median [95% CI])*	*P* value
AST (IU/L)	40.5 [22.8 to 50.4]	63.0 [37.3 to 120.1]	*P *= 0.0003
ALT (IU/L)	24.5 [17.5 to 41.9]	57 [32.2 to 72.7]	*P *= 0.0002
ALP (IU/L)	118 [101.6 to 143.7]	109 [96.3 to 119.4]	*P *= 0.012
Total bilirubin (mg/dL)	0.9 [0.7 to 1.2]	0.8 [0.6 to 1.9]	*P *= 0.03
Direct bilirubin (mg/dL)	0.4 [0.3 to 0.5]	0.4 [0.3 to 0.9]	*P *= 0.01
GGT (IU/L)	65.5 [30.7 to 88.6]	63.0 [54.2 to 86.5]	*P *= 0.7
INR	1.2 [1.1 to 1.4]	1.2 [1.1 to 1.3]	*P *= 0.9
Serum albumin (g/L)	37.6 ± 8.4	36.4 ± 6.9	*P *= 0.2
PLT (× 10^3^/μL)	89.3 ± 51.0	82.7 ± 46.8	*P *= 0.47
WBC (× 10^9^/L)	4.3 ± 1.5	6.2 ± 3.3	*P *= 0.002
Neutrophils (× 10^9^/L)	1.3 ± 3.2	0.7 ± 5.3	*P *= 0.2
% Neutrophils	56.7 ± 11.8	78.6 ± 10.0	*P *< 0.0001

*Average ± SD if the variable has a normal distribution; median [95% CI] if the variable does not have a normal distribution

*AST* aspartate transaminase; *ALT* alanine transaminase; *ALP*: alkaline phosphatase; *GGT*: gamma-glutamyl transferase; *INR* international normalized ratio; *PLT* platelets; *WBC* white blood cells

Per-patient analysis, at 1 month: in 7/22 patients (31.8%), there was a complete response (CR), and in 13/22 patients (59.1%), there was a partial response (PR) for a total objective response (OR; [CR + PR]) of 90.9% (20/22 patients). In two patients (2/22; 9.1%), we observed progressive disease (PD), due to the occurrence of a new tumour in another liver segment.

Per-procedure analysis, at 1 month: 10/24 (41.7%) were on CR, and 14/24 (58.3%) were on PR for an OR of 100% (24/24).

**Fig. 3:**
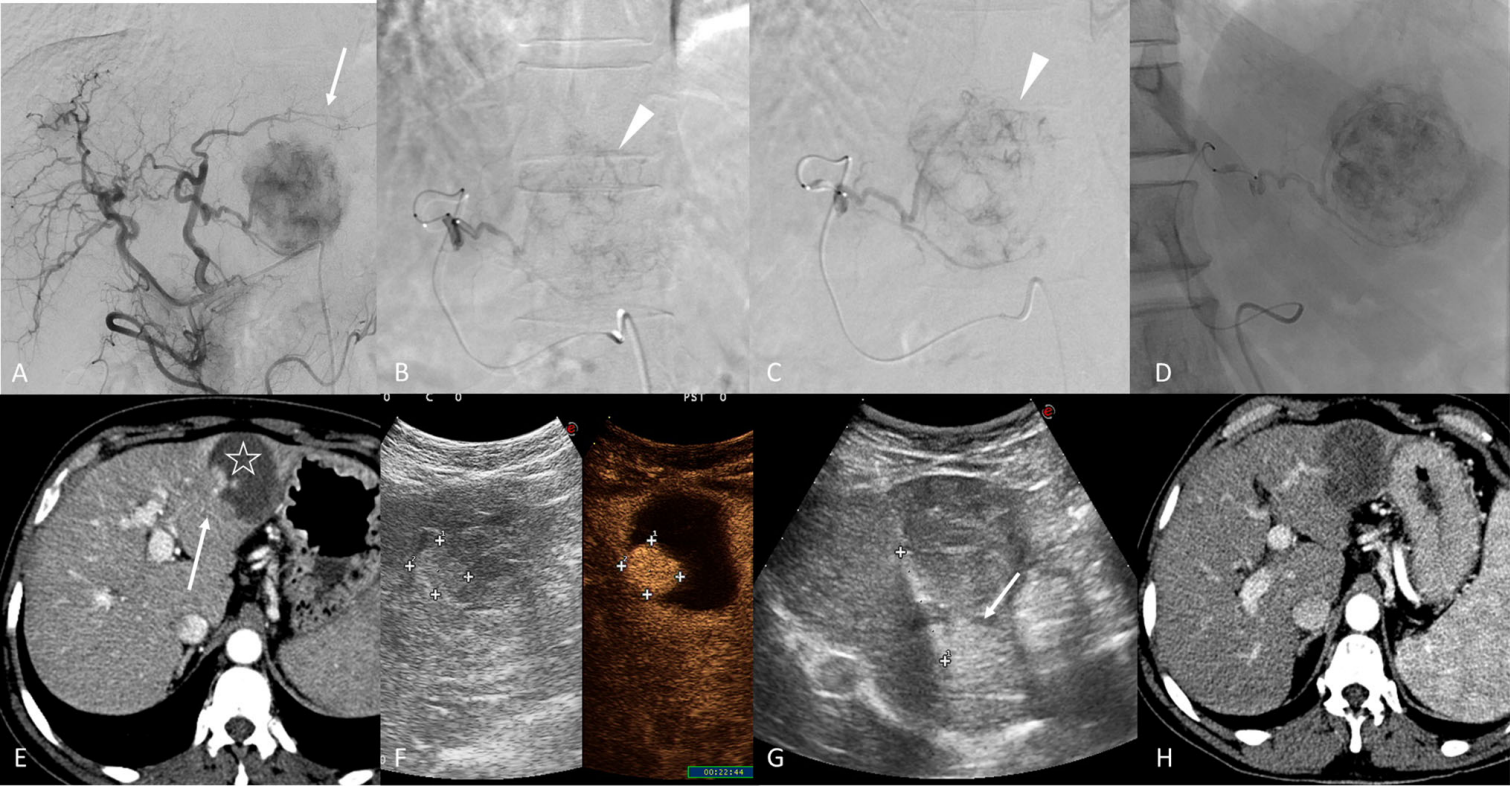
A 156-year-old male with a single nodule of HCC with maximal diameter of 76 mm at II/III hepatic segments. Figure **A**, Digital subtraction angiography (DSA) performed from the common hepatic artery, shows the hypervascular structure of the HCC in the left lobe (arrow). Super-selective DSA of the tumour with deflated balloon figure **B**) and inflated balloon figure **C**) (arrowhead). Figure **D** shows single fluoroscopy image after the embolization. Figure **E**, 1-month follow-up CT in arterial phase, highlights the partial necrosis of the nodule (star) with the presence of a hypervascular bottom (arrow) of vital residual tumour. Figure **F**, contrast-enhanced ultra-sound, shows the residual HCC, and figure **G** evidences the radiofrequency ablation of the lesion (arrow). Figure **H**, post-procedural CT in arterial phase, evidences the complete response of the HCC

**Fig. 4:**
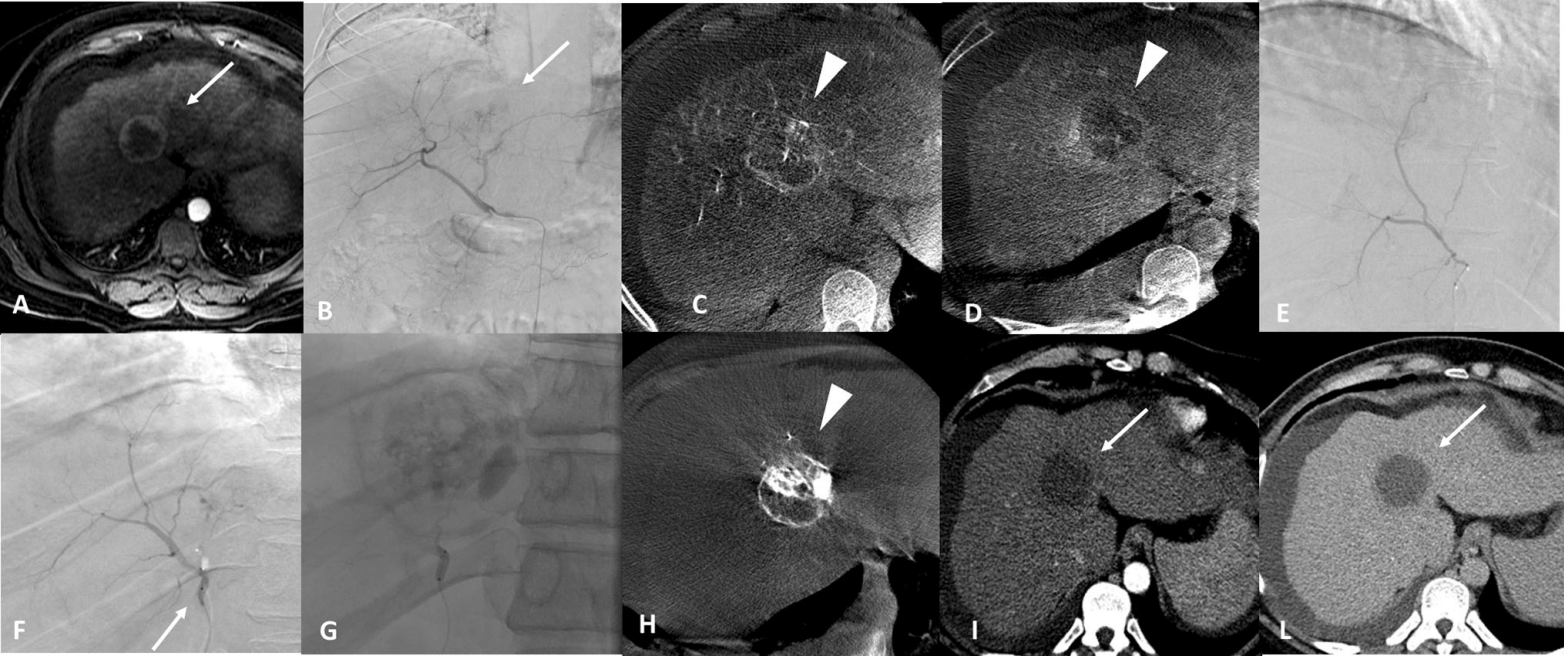
A 42-year-old female with HCC at level of the IV hepatic segment (maximal diameter 42 mm). Figure **A**, magnetic resonance imaging, arterial phase, shows non-homogeneous hypervascular nodule in the IV segment (arrow). The tumour is confirmed by the digital subtracted angiography (DSA) (figure **B**, arrow), in the cone-beam CT arterial phase (figure **C**) (arrowhead) and cone-beam CT delayed phase (figure **D**) (arrowhead). Super-selective DSA of the tumour with deflated balloon figure **E**) and inflated balloon (arrow) figure **F**). Figure **G** shows single fluoroscopy image after the embolization. Figure **H** non-enhanced cone-beam CT at the end of the procedure shows complete filling of the HCC (qTCR) (arrowhead). 1-month follow-up CT in arterial phase (figure **I**) and late phase (figure **L**) demonstrates the complete response of the HCC (arrow)

Among patients classified as partial responders, the average percentage of reduction in tumour volume was 64.9 ± 27.3% with an absolute tumour reduction volume median of 6.5 cm^3^ (95% CI 1.8 to 13.3). The baseline tumour volume was 17.7 cm^3^ (95% CI 8.3 to 38.2), and this value decreased to 1.2 cm^3^ (95% CI 0.0 to 8.8) at 1 month (< 0.05) (Figs. 3, 4).

Per-patients analysis at 3–6 months showed a CR of 14.3% (2 out of 14 patients) and a PR 42.9% (6 out 14) leading to an OR of 57.2% (8/14). The PD rate was 42.8% (6 out of 14), of these, 2/6 (33.3%) due to a new tumour in another segment.

Per-procedure analysis, at 3–6 months: 9/17 (52.9%) were CR, 4/17 (23.6%) were PR, and 4/17 (23.5%) were PD. The OR rate was 76.5% (13/17).

## Discussion

This preliminary study shows that b-TACE performed in conjunction with epirubicin-loaded DEM is a safe procedure and is associated with a high overall tumour response.

Despite the initial complexity added to the procedure by the presence of the balloon micro-catheter and the different catheter manipulation technique, the operators were able to position and inflate the balloon micro-catheter in the selected vascular segment in all cases. This is of note, considering that 70% of balloon placements were performed in a second-order hepatic artery branch or in a more distal branch. All catheterization procedures were carried out without observing a vascular complication that could stop the embolization procedure, such as vasospasm or a dissection. In all cases, the balloon micro-catheter visibility was good enough to perform procedures.

Our data on the BOASP reduction (63.2 ± 14.7 mmHg) are in line with those reported by Irie et al. [2] that identified a better lipiodol dense accumulation with a BOASP lower than 64 mmHg. In our study, we observed only one case of suboptimal reduction of the BOASP **(**prior to inflation it was 210 mmHg; after inflation it was 110 mmHg). This was probably due to the vascular anatomy of this patient which had previously undergone a right hepatectomy that limited the correct placement of the balloon micro-catheter proximal to all HCC feeders. This patient, as expected, experienced a partial response to the b-TACE procedure and required a second treatment.

Regarding the site of balloon micro-catheter placement, inflation and subsequent embolization, it is worth to underline that b-TACE was performed differently from standard DEM-TACE procedures. In fact, while DEM-TACE procedures are usually performed by embolizing “as super-selective as possible within all lesion’s feeders” (scores 0, 1), b-TACE in this study was performed more proximal (“as selective as possible proximal to all lesion’s feeders”) by placing the balloon micro-catheter proximal to all lesions’ feeders. This was done with the rationale that balloon micro-catheter inflation within the arterial segment proximal to the lesions’ feeders may determine a drop of the “vis a tergo”, confirmed by the drop of the BOASP, permitting flow redirection towards a low-resistance territory such as a hypervascular HCC [21]. The theoretical background underlying this procedural decision was to maximize the DEM deposition inside the tumour. This hypothesis is confirmed by the analysis performed at post-procedural non-enhanced CBCT that demonstrated presence of DEM within the target lesion. Moreover, despite that extra-nodular DEM deposition was seen in 54.1% of procedures, this was categorized as diffuse only in 4 cases and was not associated to post-procedural complications.

Of the four significant AEs, only the pseudo-aneurysm of the arterial feeder was strictly related to the use of the balloon micro-catheter. Pseudo-aneurysm formation has been reported and is correlated with the learning curve in the correct use of this device [22]. This episode occurred in fact within the first five cases of our experience. The remaining AEs represent a known complication of both the DEM-TACE procedures and b-TACE performed with lipiodol [15, 23].

Several aspects should be kept in mind when dilating the balloon micro-catheter: first, despite its compliant structure (the balloon is made of polyurethane), over-dilation may result in a vascular damage of small arteries; second, flushing the dead space of the micro-catheter balloon’s line is crucial because a suboptimal flushing may impair the correct visualization of the balloon inflated to its nominal diameter; third, during micro-balloon inflation continuous BOASP monitoring is suggested because pressure drop may occur even before the required balloon diameter is reached. This third point is of particular relevance because often there is no perfect match between the recommended injected volume and the actual calibre of the inflated micro-balloon. In our opinion, inflation should be always performed under fluoroscopy as the micro-balloon needs an appropriate time (longer than a standard angioplasty balloon) to reach the required diameter.

Concerning laboratory findings, all post-procedural modifications were grade 1 according to CTCAEv5.0, despite the high degree of tumour devascularization (average debulking percentage 61.4 ± 31.2%) and the fact that the embolization was segmental or even more proximal in 14/24 procedures (58.3%).

With the exception of abdominal pain (20.1% vs. 14% [23]), each aspect of PES had a lower incidence, as compared with the incidence reported in the existing literature, in particular fever was 4.0% versus 78% and 68% [23], and nausea was 20.1% versus 28% [24].

Due to the lack of data on the b-TACE performed with DEM, the comparison with the existing literature is influenced by several causes of bias: use of lipiodol, different drugs and population characteristics (e.g. number and dimension of treated tumours).

Despite these limitations, the objective response of this series is 90.9% and 58.3%, respectively, at 1 and 3–6 months which are consistent with the results reported by Hatanaka et al. [6] (OR of 63.6%), using miriplatin with lipiodol, and Kawamura et al. [25], Asayama et al. [26] and Minami et al. [23], reporting an OR of 59.6%, 57.1% and 56.3%, respectively. This consistency is particularly relevant as our cohort of patients presented greater tumour comparing with other studies (max diameter 32.1 mm ± 14.4 [range 12–64 mm] versus 2.0 mm ± 0.9 [23] versus 6.6 mm [range 9–40 mm] [26]). Further studies, with randomized control group, are necessary to deeply investigate this aspect.

Partial correlation analysis results between BOASP during balloon inflation and percentage volume reduction, categorized for tumour < 3 and > 3 cm (*r* = − 0.05; − 0.46; *P *> 0.05, respectively), demonstrated a moderate inverse correlation in the subset of patients having tumours > 3 cm as confirmed in Fig. 2. This finding suggests a potential major benefit of the b-TACE procedure in the cohort of patients having tumour > 3 cm.

This is of particular relevance considering the initial indication for b-TACE. Four out of five patients (80%) with a total tumour burden outside the “up-to-seven” criteria for liver transplantation [27] were down-staged and subsequently listed in the active waiting list for liver transplantation. Among these four patients, in three cases a tumour greater than 5 cm was treated with a b-TACE as a standalone treatment which achieved complete necrosis, whereas the fourth patient with a tumour greater than 5 cm, b-TACE achieved effective tumour debulking and allowed the execution of adjunctive RFA of the residual vital tissue.

Our study presents several limitations, first the small sample size sample size (22 patients), second, the non-randomized nature of this series.

In conclusion, our series showed that the combination of b-TACE and DEM was safe and obtained a CR, at 1 and 3–6 months, of 41.7% (10/24) and 52.9% (9/17) and a PR of 58.3% (14/24) and 4/17 (23.5%), respectively.

## References

[1] European Association for the Study of The L, European Organisation for R, Treatment of C (2012). EASL–EORTC clinical practice guidelines: management of hepatocellular carcinoma. J Hepatol.

[2] Irie T, Kuramochi M, Takahashi N (2013). Dense accumulation of lipiodol emulsion in hepatocellular carcinoma nodule during selective balloon-occluded transarterial chemoembolization: measurement of balloon-occluded arterial stump pressure. Cardiovasc Interv Radiol.

[3] Sugimoto K, Saguchi T, Saito K, Imai Y, Moriyasu F (2014). Hemodynamic changes during balloon-occluded transarterial chemoembolization (b-TACE) of hepatocellular carcinoma observed by contrast-enhanced ultrasound. J Med Ultrason.

[4] Arai H, Abe T, Takayama H (2015). Safety and efficacy of balloon-occluded transcatheter arterial chemoembolization using miriplatin for hepatocellular carcinoma. Hepatol Res.

[5] Maruyama M, Yoshizako T, Nakamura T, Nakamura M, Yoshida R, Kitagaki H (2016). Initial experience with balloon-occluded trans-catheter arterial chemoembolization (B-TACE) for hepatocellular carcinoma. Cardiovasc Interv Radiol.

[6] Hatanaka T, Arai H, Kakizaki S (2018). Balloon-occluded transcatheter arterial chemoembolization for hepatocellular carcinoma. World J Hepatol.

[7] Irie T, Kuramochi M, Kamoshida T, Takahashi N (2016). Selective balloon-occluded transarterial chemoembolization for patients with one or two hepatocellular carcinoma nodules: retrospective comparison with conventional super-selective TACE. Hepatol Res.

[8] Ogawa M, Takayasu K, Hirayama M (2016). Efficacy of a microballoon catheter in transarterial chemoembolization of hepatocellular carcinoma using miriplatin, a lipophilic anticancer drug: short-term results. Hepatol Res.

[9] Lammer J, Malagari K, Vogl T (2010). Prospective randomized study of doxorubicin-eluting-bead embolization in the treatment of hepatocellular carcinoma: results of the PRECISION V study. Cardiovasc Interv Radiol.

[10] Golfieri R, Giampalma E, Renzulli M (2014). Randomised controlled trial of doxorubicin-eluting beads versus conventional chemoembolisation for hepatocellular carcinoma. Br J Cancer.

[11] Sacco R, Bargellini I, Bertini M (2011). Conventional versus doxorubicin-eluting bead transarterial chemoembolization for hepatocellular carcinoma. J Vasc Interv Radiol JVIR.

[12] Choi JY, Lee JM, Sirlin CB (2014). CT and MR imaging diagnosis and staging of hepatocellular carcinoma: part I. Development, growth, and spread: key pathologic and imaging aspects. Radiology.

[13] Bruix J, Sherman J, American Association for the Study of Liver D (2011). Management of hepatocellular carcinoma: an update. Hepatology.

[14] Ponziani FR, Zocco MA, Campanale C (2010). Portal vein thrombosis: insight into physiopathology, diagnosis, and treatment. World J Gastroenterol.

[15] Lucatelli P (2018). Polyethylene glycol (PEG)-epirubicin loaded drug-elunting embolic (DEE)-TACE procedures utilizing a combined approach with 100- and 200- µm microspheres. Iniatial Exp J Vasc Interv Radiol.

[16] Wang X, Erinjeri JP, Jia X (2013). Pattern of retained contrast on immediate postprocedure computed tomography (CT) after particle embolization of liver tumors predicts subsequent treatment response. Cardiovasc Interv Radiol.

[17] Lencioni R, Llovet JM (2010). Modified RECIST (mRECIST) assessment for hepatocellular carcinoma. Semin Liver Dis.

[18] Filippiadis DK, Binkert C, Pellerin O, Hoffmann RT, Krajina A, Pereira PL (2017). Cirse quality assurance document and standards for classification of complications: the cirse classification system. Cardiovasc Interv Radiol.

[19] Services USDOHAH (2017) Common Terminology Criteria for Adverse Events (CTCAE) Version 5.0.

[20] Wang Z, Wang M, Duan F, Song P, Liu F (2014). Bile duct injury after transcatheter arterial chemoembolization: risk factors and clinical implications. Hepato-Gastroenterol.

[21] Aramburu J, Anton R, Rivas A (2018). Numerical zero-dimensional hepatic artery hemodynamics model for balloon-occluded transarterial chemoembolization. Int J Numer Methods Biomed Eng.

[22] Matsumoto T, Endo J, Hashida K (2015). Balloon-occluded transarterial chemoembolization using a 1.8-French tip coaxial microballoon catheter for hepatocellular carcinoma: technical and safety considerations. Minim Invasive Therapy Allied Technol (MITAT).

[23] Minami Y, Minami T, Chishina H (2015). Balloon-occluded transcatheter arterial chemoembolization for hepatocellular carcinoma: a single-center experience. Oncology.

[24] Ishikawa T, Abe S, Inoue R (2014). Predictive factor of local recurrence after balloon-occluded TACE with miriplatin (MPT) in hepatocellular carcinoma. PLoS ONE.

[25] Kawamura Y, Ikeda K, Fujiyama S (2017). Usefulness and limitations of balloon-occluded transcatheter arterial chemoembolization using miriplatin for patients with four or fewer hepatocellular carcinoma nodules. Hepatol Res.

[26] Asayama Y, Nishie A, Ishigami K (2016). Hemodynamic changes under balloon occlusion of hepatic artery: predictor of the short-term therapeutic effect of balloon-occluded transcatheter arterial chemolipiodolization using miriplatin for hepatocellular carcinoma. SpringerPlus.

[27] Duffy JP, Vardanian A, Benjamin E (2007). Liver transplantation criteria for hepatocellular carcinoma should be expanded: a 22-year experience with 467 patients at UCLA. Ann Surg.

